# Network-dosage compensation topologies as recurrent network motifs in natural gene networks

**DOI:** 10.1186/1752-0509-8-69

**Published:** 2014-06-14

**Authors:** Ruijie Song, Ping Liu, Murat Acar

**Affiliations:** 1 Interdepartmental Program in Computational Biology and Bioinformatics, Yale University, 300 George Street, Suite 501, New Haven, CT 06511, USA; 2 Department of Molecular, Cellular and Developmental Biology, Yale University, 219 Prospect Street, P.O. Box 27391, New Haven, CT 06511, USA; 3 Systems Biology Institute, Yale University, 840 West Campus Drive, West Haven, CT 06516, USA

**Keywords:** Network-dosage compensation, Network motifs, Yeast galactose network, Stochasticity, Genetic interactions

## Abstract

**Background:**

Global noise in gene expression and chromosome duplication during cell-cycle progression cause inevitable fluctuations in the effective number of copies of gene networks in cells. These indirect and direct alterations of network copy numbers have the potential to change the output or activity of a gene network. For networks whose specific activity levels are crucial for optimally maintaining cellular functions, cells need to implement mechanisms to robustly compensate the effects of network dosage fluctuations.

**Results:**

Here, we determine the necessary conditions for generalized N-component gene networks to be network-dosage compensated and show that the compensation mechanism can robustly operate over large ranges of gene expression levels. Furthermore, we show that the conditions that are necessary for network-dosage compensation are also sufficient. Finally, using genome-wide protein-DNA and protein-protein interaction data, we search the yeast genome for the abundance of specific dosage-compensation motifs and show that a substantial percentage of the natural networks identified contain at least one dosage-compensation motif.

**Conclusions:**

Our results strengthen the hypothesis that the special network topologies that are necessary for network-dosage compensation may be recurrent network motifs in eukaryotic genomes and therefore may be an important design principle in gene network assembly in cells.

## Background

The effective dosage of a gene network – the number of network copies in a cell – can vary significantly both throughout a cell’s lifetime and across different cells in the same clonal population. Such changes can arise from a variety of direct and indirect causes. For example, DNA replication during the cell cycle [1] would double the network dosage, and it has been shown that many promoters indeed display an increase in transcription consistent with gene dosage effects during the G2 phase of the cell cycle as compared to G1 [2]. Similarly, organisms such as yeast that switch between haploid and diploid life forms [3] must cope with the increased network dosage. Global noise in gene expression [4,5] could also lead to significant variations in effective network dosage. Moreover, such changes can have significant effects on the cellular phenotypes [6]. For example, in multicellular organisms, widespread dosage changes can be fatal [7]. It stands to reason, therefore, that cells must have evolved mechanisms to compensate for such dosage alterations, including the inevitable alterations occurring at the gene network level. Despite the presence of previous studies [8,9] focusing on dosage variations of individual genes, there is very little work [10] approaching this question from the gene network point of view. Due to the presence of nonlinear feedback interactions among the individual genes of a gene network, dosage compensation analyses focusing on individual genes one at a time cannot reliably predict whether or not the activity of their network would be compensated.

A previous study [10] has demonstrated that the galactose signaling pathway (GAL pathway) in *Saccharomyces cerevisiae* is dosage compensated on the network level: the activity of the network showed no significant change when the dosage of the entire regulatory network was halved in diploid cells. By mathematically and computationally analyzing 2-component networks, the study further demonstrated that such compensation effect could arise solely as a feature of the structure of the gene network. Outside of a trivial case, 1-component networks could not be dosage invariant, but 2-component networks could be if they satisfied certain criteria: the two components had to have different regulatory signs, they had to interact with a 1:1 stoichiometry, and the effects of one of the two components had to be indirect and exerted its effects on transcription through action on the other component [10].

The questions remain, however, regarding how the network-dosage compensation analysis can be extended to N-component networks, whether the compensation mechanism can robustly operate over broad gene expression ranges, and finally whether the specific dosage-compensation topologies are recurrent network motifs in natural gene networks. In this study, we first expand the mathematical compensation analysis beyond the 1-component and 2-component networks, so that the analysis includes gene networks of any size (N-component networks), demonstrating that a necessary condition for dosage compensation in such networks is the existence of a 2-component subnetwork with an activator and an inhibitor. We then perform a network-dosage compensation analysis on 2-component networks and show that the compensation mechanism acts over large gene expression and protein degradation ranges, not just the specific levels displayed by the GAL network components. Furthermore, we show that the conditions that are necessary for an inducible network to be network-dosage compensated are also sufficient. Finally, using genome-wide protein-DNA and protein-protein interaction data, we search the *S. cerevisiae* genome for the abundance of the special network topologies necessary for network-dosage compensation, and show that a substantial percentage of the natural networks identified contain at least one dosage-compensation topology.

## Results

### Mathematical analysis of network-dosage compensation in N-component gene networks

To investigate the necessary and sufficient conditions that can make an N-component gene network dosage-compensated, we consider a network composed of N genes that are under the control of the same transcription factor (TF). In our analysis, we define gene networks as structures that have varying numbers of genes that are all under the control of a common TF for each gene network.

The network under consideration can be represented by the following set of differential equations describing the time evolution of the concentrations of proteins expressed from the network genes:

(1) $$\left\{\begin{array}{c} \frac{dx_{1}}{dt}=\theta_{1}f\left(\rho ,x_{1},\ldots ,x_{N}\right)-\gamma x_{1}\\ \vdots \\ \frac{dx_{N}}{dt}=\theta_{N}f\left(\rho ,x_{1},\ldots ,x_{N}\right)-\gamma x_{N} \end{array}\right)$$

Here, *x*_*i*_ represents the average total concentration of the *i*^*th*^ protein, *θ*_*i*_ represents the maximal expression rate for the *i*^*th*^ gene, *γ* represents the cell-division rate, *ρ* represents an external control parameter with which the network can be induced, and *f(ρ,x*_*1*_*,…,x*_*N*_*)* represents the activity of the gene network or the fraction of active promoter sites. We assume that the network proteins are diluted at the cell-division rate (*γ*), corresponding to cases in which protein lifetimes are much longer than the cell-division time. We further assume that network proteins interact with each other on fast timescales and that these interactions determine the fractional activity of the transcription center represented by the function *f,* whose value is limited to the range [0, 1]. Then, each gene is expressed proportionally to the activity of the common transcription center.

With this framework, we are interested in elucidating the general network features that can keep the activity of the transcriptional center to be compensated (or invariant) to parallel changes in the maximal expression rate of the network genes. Cells would experience such parallel changes due to the effects of global noise in expression, or when chromosomes are replicated during cell cycle progression.

From Eq. 1, we know that at steady state

(2) $$\frac{\theta_{1}}{x_{1}}=\frac{\theta_{2}}{x_{2}}=\ldots =\frac{\theta_{N}}{x_{N}}=\frac{\gamma }{f\left(\rho ,x_{1},x_{2},\ldots ,x_{N}\right)}$$

We consider cases in which *θ*_*1*_*, θ*_*2*_*, …, θ*_*N*_ are proportionally changed by introducing a new parameter:

(3) $$\left\{\begin{array}{c} \left(1+\delta \right)\theta_{1}f\left(\rho ,x_{1},\ldots ,x_{N}\right)=\gamma x_{1}\\ \vdots \\ \left(1+\delta \right)\theta_{N}f\left(\rho ,x_{1},\ldots ,x_{N}\right)=\gamma x_{N} \end{array}\right)$$

Taking the derivative of both sides of the first equation above with respect to *δ*, we have

(4) $$\theta_{1}f+\left(1+\delta \right)\theta_{1}\left(\frac{\partial f}{\partial x_{1}}\frac{dx_{1}}{d\delta }+\ldots +\frac{\partial f}{\partial x_{N}}\frac{dx_{N}}{d\delta }\right)=\gamma \frac{dx_{1}}{d\delta }$$

From Eq. 2, we have

$$x_{m}=\frac{\theta_{m}}{\theta_{1}}x_{1}\;\mathrm{for}\;m=2,\ldots ,N$$

Therefore,

(5) $$\frac{dx_{m}}{d\delta }=\frac{\theta_{m}}{\theta_{1}}\frac{dx_{1}}{d\delta }\;\mathrm{for}\;m=2,\ldots ,N$$

Plugging this equation into Eq. 4, we obtain an equation that can be solved for $\frac{dx_{1}}{d\delta }$:

(6) $$\frac{dx_{1}}{d\delta }=\frac{\theta_{1}f}{\gamma -\left(1+\delta \right)\left(\theta_{1}\frac{\partial f}{\partial x_{1}}+\ldots +\theta_{N}\frac{\partial f}{\partial x_{N}}\right)}$$

at steady state. Combining Eq. 5 and Eq. 6 for *m = 2, …, N*, we have

(7) $$\frac{dx_{m}}{d\delta }=\frac{\theta_{m}f}{\gamma -\left(1+\delta \right)\left(\theta_{1}\frac{\partial f}{\partial x_{1}}+\ldots +\theta_{N}\frac{\partial f}{\partial x_{N}}\right)}$$

Therefore,

(8) $$\frac{df}{d\delta }=\sum_{i=1}^{N}\frac{\partial f}{\partial x_{i}}\frac{dx_{i}}{d\delta }=\frac{\left(\theta_{1}\frac{\partial f}{\partial x_{1}}+\ldots +\theta_{N}\frac{\partial f}{\partial x_{N}}\right)f}{\gamma -\left(1+\delta \right)\left(\theta_{1}\frac{\partial f}{\partial x_{1}}+\ldots +\theta_{N}\frac{\partial f}{\partial x_{N}}\right)}$$

For $\frac{df}{d\delta }$ to be zero with generality, we must have $\theta_{1}\frac{\partial f}{\partial x_{1}}+\ldots +\theta_{N}\frac{\partial f}{\partial x_{N}}=0$. As all parameters here are positive and at least some of the partial derivatives are nonzero, at least one of the partial derivatives must be positive and at least one must be negative. Therefore, in order to keep the activity of an N-component gene network compensated against parallel changes in the number of network components, a necessary condition is that the network has to be composed of components of different regulatory signs (*e.g.* 1 activator and N-1 inhibitors, 2 activators and N-2 inhibitors, etc.). In other words, the gene network must have a 2-component *subnetwork* with components of different regulatory signs (*i.e.,* one activator and one inhibitor).

How can certain interaction topologies between network components facilitate the dosage compensation behavior of the network activity? Will the compensation mechanism operate for a wide range of gene expression levels and protein degradation rates, or is it limited to the specific parameter values used in the previous work [10], which correspond to the GAL network? 2-component *subnetworks* composed of one activator and one inhibitor provide effective minimal systems to address these questions. In the next section, we numerically analyze 2-component *subnetworks* to find out whether or not specific gene expression and protein degradation levels are required for observing compensated network activity in gene networks.

### Sensitivity analysis of the network-dosage compensation mechanism with respect to the gene expression and protein degradation levels

**Figure 1:**
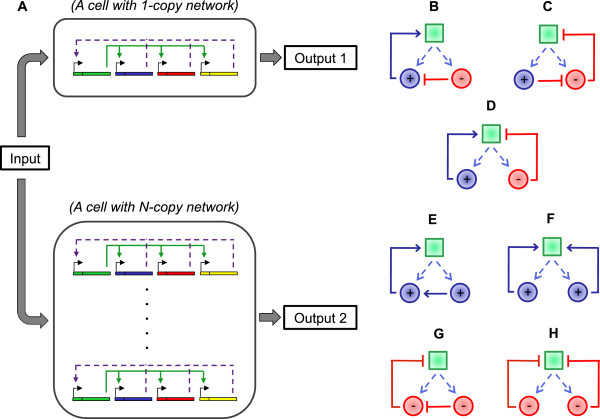
**Network-dosage compensation and specific topologies that are capable or incapable of facilitating network-dosage compensation. A**. Cells having one- or N-copies of a general gene network. Each copy of the network is composed of a master transcription factor (encoded by the first gene, in green) of the cascade and additional components (blue, red, yellow genes driven by their own promoters carrying binding sites for the common TF). In turn, the expression levels of the additional network components can affect the activity of the network (purple dashed arrows). The network(s) in each cell is induced by an input signal. Depending on whether or not the network has the dosage-compensation capability, the two outputs can be similar or different. **B-H**. Dosage-compensated and non-compensated network structures. Green squares represent the transcriptional machinery that controls the expression of the two network components. **B-C**. Two network structures that are capable of dosage compensation. **D**. A network structure that is incapable of dosage compensation. **E-H**. Four network structures that could not be dosage compensated because the two network components have the same regulatory sign.

To explore if certain 2-component interaction topologies would make it easier or harder for cells to show network dosage compensation, we numerically analyzed 2-component topologies in which an activator (*a*) and an inhibitor (*i*) are controlled by a common transcriptional center and quantified their compensation and inducibility levels. The specific interaction schemes we analyzed are depicted in Figure 1B-D. Each interaction topology is represented by a mathematical form involving four parameters quantifying the scales of action for the activator (*S*_*a*_) and inhibitor (*S*_*i*_) and the nonlinearities with which the activator (α) and inhibitor (β) interacts with their downstream targets, as follows (Additional file 1: Figure S2):Topology in Figure 1B:

(9) $$f\left(g,a,i\right)=\frac{1}{1+{\left(\frac{S_{a}\mathrm{ga}}{1+{\left(S_{i}i\right)}^{\beta }}\right)}^{-\alpha }}$$

Topology in Figure 1C:

(10) $$f\left(g,a,i\right)=\frac{1}{1+{\left(\frac{S_{i}i}{1+{\left(S_{a}\mathrm{ga}\right)}^{\alpha }}\right)}^{\beta }}$$

Topology in Figure 1D:

(11) $$f\left(g,a,i\right)=\frac{1}{1+{\left(S_{a}\mathrm{ga}\right)}^{-\alpha }}\cdot \frac{1}{1+{\left(S_{i}i\right)}^{\beta }}$$

For each topology, our analysis involved numerically solving the following differential equations at t = 24 h:

(12) $$\left\{\begin{array}{c} \frac{\mathrm{da}}{\mathrm{dt}}=N\theta_{a}\left[\lambda_{a}\left(1-f\left(g,a,i\right)\right)+f\left(g,a,i\right)\right]-\gamma_{0}a-\gamma_{a}a\\ \frac{\mathrm{di}}{\mathrm{dt}}=N\theta_{i}\left[\lambda_{i}\left(1-f\left(g,a,i\right)\right)+f\left(g,a,i\right)\right]-\gamma_{0}i-\gamma_{i}i \end{array}\right)$$

In the mathematical form describing *f*, the parameter values corresponding to *S*_*a*_, *S*_*i*_, *α* and *β* were sampled from large ranges as described in Table 1. To verify the generality of the compensation mechanism beyond the specific context of the GAL network [10], we chose a variety of different values for the parameters describing the maximal transcription/translation activity (*θ*_*a*_ and *θ*_*i*_) and the rate of protein degradation (*γ*_*a*_ and *γ*_*i*_) (Table 1). In these equations, *γ*_*0*_ is the cell division rate and *λ*_*a*_ and *λ*_*i*_ quantifies the basal protein expression level. Inserted into the above coupled differential equation, each set of the sampled parameters, the chosen parameters, and the external inducer level (*g*) corresponded to a new solution for [*a, i*]. We used the resulting values for activator and inhibitor concentrations to obtain numerical inducibility curves, defined for each topology by *f(g, a, i).*

**Table 1:** List of ODE model parameters, their descriptions, and values

**Parameter**	**Description**	**Value**
S_a_	Activator scale of action	Logarithmically sampled from [10^−3^, 10^3^]
S_i_	Inhibitor scale of action	Logarithmically sampled from [10^−4^, 10^2^]
α	Stoichiometry parameter	Linearly sampled from [0.2, 5]
β	Stoichiometry parameter	Linearly sampled from [0.2, 5]
θ_a_	Activator production rate	300, 1500 or 7500/hr
θ_i_	Inhibitor production rate	300, 1500 or 7500/hr
γ_0_	Dilution rate constant	0.46/hr
t_a_	Activator half-life	5, 30, 120 or ∞ min
γ_a_	Activator degradation rate constant	ln(2)/t_a_
t_i_	Inhibitor half-life	5, 30, 120 or ∞ min
γ_i_	Inhibitor degradation rate constant	ln(2)/t_i_
N	Network copy number	1 or 2
λ_a_	Activator basal production coefficient	0.20
λ_i_	Inhibitor basal production coefficient	0.20
g	Inducer strength	10^-2+0.025C^, where C = 0, 1, 2, …, 80

To quantify the degree of compensation in each network topology, we produced separate inducibility curves with one (N = 1) or two (N = 2) sets of the activator and inhibitor genes, and computed the area between those curves. The larger the area between the two curves, the higher the penalty to compensation in the network (Additional file 1: Figure S1A). In principle, dosage-varied networks that cannot be activated beyond their basal activity levels or networks that always stay ‘ON’ irrespective of the inducer levels can also be classified as dosage-compensated, but they lack the ability to act as regulatory networks against external physiological signals. Therefore, it is also important to determine if a dosage-compensated network’s inducibility level corresponds to physiologically relevant levels. For this, we quantified the relative inducibility levels of the numerical inducibility curves against a reference inducibility curve (Additional file 1: Figure S1B and C), and plotted them against the compensation levels. Representative plots for a random sample containing approximately 1.4% of the networks examined (20,000 networks out of 1,440,000) are presented in Additional file 1: Figure S2D-F. We found that only networks with the topologies in Figure 1B and C are capable of showing simultaneously high degrees of dosage-compensation and inducibility.

**Figure 2:**
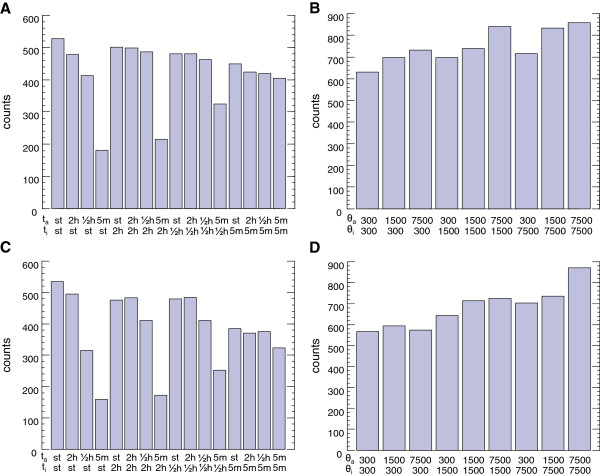
**The compensation mechanism can operate over large ranges of protein production and degradation rates. A**. Bar plots showing the number of inducible and dosage compensated networks (N = 6746) having the topology of Figure 1B with the specified combination of protein half-lives. st, stable. **B**. Bar plots showing the number of inducible and dosage compensated networks having the topology of Figure 1B with the specified combination of maximum protein production rates (in proteins/hour) θ_a_ and θ_i_. **C**. Bar plots showing the number of inducible and dosage compensated networks (N = 6124) having the topology of Figure 1C with the specified combination of protein half-lives. st, stable. **D**. Bar plots showing the number of inducible and dosage compensated networks having the topology of Figure 1C with the specified combination of maximum protein production rates (in proteins/hour) θ_a_ and θ_i_.

To better understand the distribution of parameters that can give rise to high degrees of compensation and inducibility, we analyzed the system parameters for all networks that are both dosage compensated and inducible. This analysis included examination of the effect of protein expression levels on the compensation behavior of each topology by looking at the maximal protein production rates *θ* and protein degradation rates *γ*. To conduct the compensation analysis at different expression levels, we selected parameter values for *θ* and *γ* from large ranges that were physiologically relevant (Table 1). As shown in Figure 2, the different combinations of the values we used to run our simulations did not end up significantly affecting the population of data points falling into the compensated and inducible region of each dot plot (Additional file 1: Figure S2D-F). In other words, the compensation mechanism can robustly operate over large expression ranges and it is not limited to the expression values displayed by the GAL network components.

**Figure 3:**
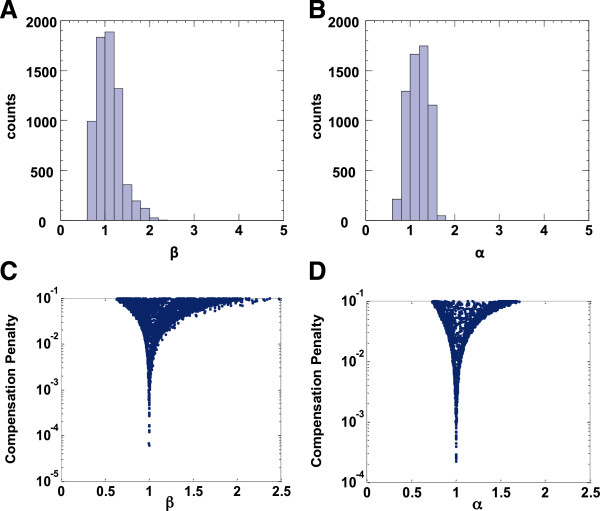
**The parameter quantifying the nonlinearity of interaction between the network components is strongly constrained in dosage-invariant inducible networks. A**. Histogram of the value of the sampled parameter β in dosage-invariant inducible networks having the topology of Figure 1B (green area in Figure S2D, N = 6746). **B**. Histogram of the value of sampled parameter α in dosage-invariant inducible networks having the topology of Figure 1C (green area in Figure S2E, N = 6124). **C-D**. Plot of the network compensation penalty versus the value of the strongly constrained network parameter, for networks that are both inducible and dosage invariant.

Analysis of the other parameters to the model demonstrate that, for each network topology, only the value of one parameter is strongly constrained in networks that are both dosage compensated and inducible (Figure 2, Figure 3A and B, Additional file 1: Figure S3). For both network topologies, the critical parameter is the parameter defining the nature of the stoichiometric interaction between the activator and inhibitor of the 2-component subnetwork (*β* for the network topology in Figure 1B and *α* for the network topology in Figure 1C), and in both topologies its values are tightly distributed around 1 (Figure 3A and B). Plotting the penalty to compensation against the strongly constrained parameter further confirms that having the value of the parameter to be very close to 1 is necessary for a small compensation penalty in an inducible network (Figure 3C and D).

### Sufficiency analysis of the network-dosage compensation conditions for well-behaved gene networks

**Figure 4:**
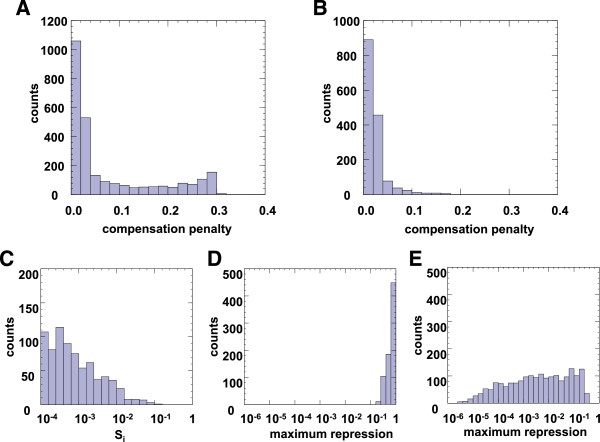
**Sufficiency analysis of the network-dosage compensation conditions. A-B**. Histogram of compensation penalty of networks with inducibility penalty < = 0.1 and constrained parameter value between 0.9 and 1.1, for the topology shown in Figure 1B **(A)** and Figure 1C **(B)**. **C**. Values of S_i_ for networks in part A with compensation penalty > 0.1. **D**. Maximum steady state repression in networks with high compensation penalties. Value of 1 means no repression; smaller value means stronger repression. **E**. Maximum steady state repression in dosage compensated networks.

The analysis above shows that having one of the two network topologies shown in Figure 1B and C, as well as a 1:1 stoichiometric interaction between the activator and the inhibitor, are necessary conditions for a network to be both inducible and dosage-compensated. The question remains, however, whether these conditions are sufficient for an inducible network to be network-dosage compensated. To answer this question, we examined the compensation penalty of all networks whose constrained parameter (*α* or *β*) is in the range [0.9, 1.1], and whose inducibility penalty is below 0.10.As shown in Figure 4B, of the examined networks with the topology in Figure 1C, 97.6% have a compensation penalty below 0.10, and 99.3% have a compensation penalty below 0.15. However, approximately 28.3% of the examined networks with the topology in Figure 1B have compensation penalties above 0.10, with 23% above 0.15 (Figure 4A), necessitating a more detailed examination of those networks.

Further examining those networks, we found that the networks with high compensation penalties uniformly have very small values of *S*_*i*_, the parameter representing the strength of the inhibitor (Figure 4C). Moreover, the maximum inhibitory effect achievable in such systems is very small (Figure 4D) compared to those in compensated systems (Figure 4E). In short, the inhibitory component of these networks is very weak, such that 1 + (*S*_*i*_*i*)^*β*^ ≈ 1 and the networks become essentially 1-component networks with a single direct activator. Such networks can still be fully inducible, but they cannot be dosage compensated [10]. Moreover, an inhibitor with so weak an inhibitory effect is unlikely to play any significant part in the output of any naturally occurring gene network.

We define a *well-behaved gene network* as a gene network that satisfies the following two conditions: 1) the network is inducible and 2) the activating or inhibiting effect of each network component on overall network output is substantial, *i.e.,* where the approximations 1 + (*S*_*i*_*i*)^*β*^ ≈ 1 and 1 + (*S*_*a*_*a*)^*α*^ ≈ 1 do *not* hold. The above analysis supports the conclusion that for a well-behaved 2-component subnetwork to show dosage compensation, it is sufficient that the subnetwork has a topology shown in Figure 1B or C, and that the stoichiometry between the activator and the inhibitor is 1:1. As naturally occurring gene regulatory networks are very likely to be well-behaved, if a natural gene network satisfies these two requirements, it would be expected to be network-dosage compensated.

### Recurrent nature of the dosage-compensation motifs in the *Saccharomyces cerevisiae* genome

**Figure 5:**
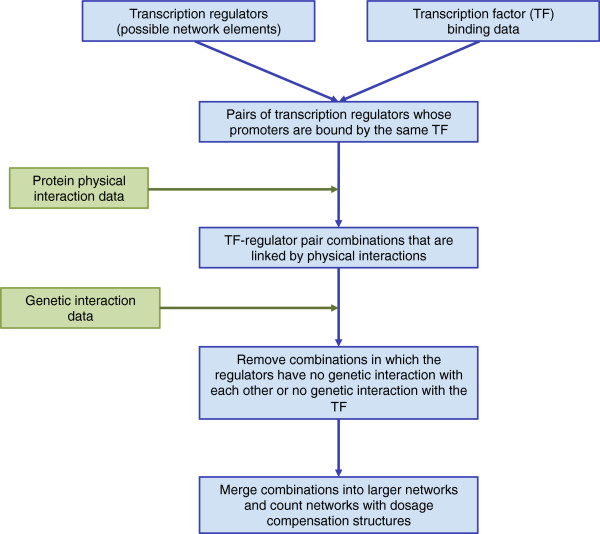
**Overview of the algorithm employed to search in the yeast genome for the abundance of the special topologies necessary for network-dosage compensation.** The algorithm takes as input a set of precompiled possible network elements (genes that potentially have a role in transcription regulation within each network), transcription factor binding data for the promoter region of each element, and physical and genetic interaction data among the elements and the transcription factors. The algorithm first searches for all possible two-component networks in which both elements are under the control of the same TF, and could influence their own expression by interacting with that TF directly or indirectly. Then, using the genetic interaction data, it attempts to determine whether the two components are of different regulatory signs and then reach a definitive conclusion on the presence of a dosage compensation motif for each TF-network combination based on the topology of the physical interaction and the regulatory sign. In post-processing, combinations for which a definitive conclusion can be made are manually combined into larger networks and the dosage compensated structures in each are counted.

To find out how frequently the dosage compensation structures occur in the yeast genome, we examined a set of 1,385 genes that have regulatory roles in *S. cerevisiae*, and 166 transcription factors (TF) that, in turn, regulate their transcription (Figure 5 and Additional files 2, 3). We limited our search to regulators because our dosage compensation structure requires both network components to affect their own transcription, directly or indirectly.

Since the network components in our dosage compensation structures are expected to affect the activity of the TF, they should physically interact with the TF either directly or indirectly. Moreover, since they are regulatory components of this network, they should genetically interact with each other, and with the TF. Therefore, we defined a potentially compensated unit (PCU) to be composed of two regulators and a TF that binds to the promoter of both of them, and in which 1) at least one regulator has a physical interaction with the TF, 2) each regulator has a reported physical interaction with either the TF or the other regulator, 3) the two regulators have at least one reported genetic interaction, and 4) at least one regulator has a reported genetic interaction with the TF.

Using this definition, we enumerated all possible PCUs using TF-DNA binding and physical and genetic interaction data (Methods, Figure 5). PCUs whose components involve global regulators, which does not regulate a small set of genes or process, were excluded. This resulted in a list of 82 PCUs, involving 57 unique regulators and 23 unique TFs (one regulator can be part of many PCUs) (Additional file 4).We then determined if the two regulators in each PCU have different regulatory signs, as required by the dosage compensation structure. Since the network structure requires the regulators to be co-expressed, we are not able to use gene expression data. Instead, we examined the documented genetic interactions between the two regulators. If the genetic interaction suggests that one regulator could compensate for the deletion of the other, we interpreted this as their having the same regulatory sign; conversely, if deletion of one regulator could compensate for the deletion of the other, then the two regulators would have different regulatory signs. Further, we looked at the physical interactions in each PCU to ensure that only one regulator physically interacts with the TF, as required by the topologies (Figure 1B-C).

**Figure 6:**
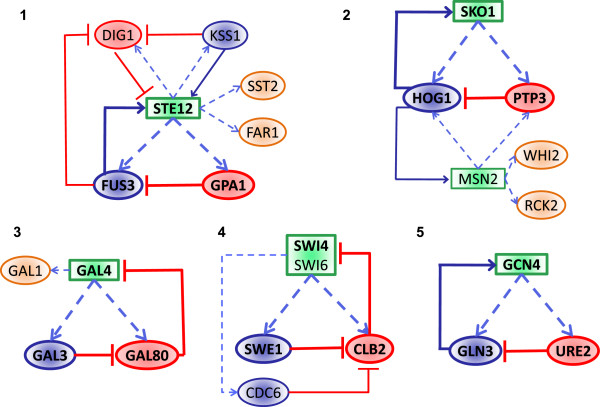
**Illustration of networks 1–5 in Table **2**.** A network-dosage compensation motif in each network (the same motif noted in Table 2) is highlighted using bolded font and borders. If a network contains more than one such motif, only one is highlighted.

In many cases, a regulator was part of multiple PCUs, some compensated and others non-compensated, because we enumerated all possible combinations. In addition, we observed several instances of false positives in PCUs reported to be potentially compensated. To get a more accurate picture of the prevalence of dosage compensation structures, we manually combined the 82 PCUs into 15 larger networks (Table 2) and verified the dosage compensation structure against the literature to ensure that the topology requirements are satisfied. Out of those 15 networks, we found that 5 networks have at least one verified dosage compensation structure (Figure 6). One of the five we found is the GAL network [10], while the others were related to pheromone response [11], response to osmotic stress [12], cell cycle control [13], and nitrogen catabolite repression [14], respectively.

**Table 2:** List of the 15 larger networks resulting from merging the PCUs

**Network**	**Genes and TFs**	**References**
**1**	**STE12,***FUS3, GPA1*, KSS1, DIG1, SST2, FAR1	[11,15]
**2**	**SKO1***, PTP3, HOG1*, MSN2, WHI2, RCK2	[12,16]
**3**	**GAL4***, GAL3, GAL80,* GAL1	[10]
**4**	**SWI4,***SWE1, CLB2*, SWI6, CDC6	[13,17]
**5**	**GCN4***, GLN3, URE2*	[14,18,19]
6	RAP1, TEL1, RIF2	
7	INO4, INO2, TYE7	
8	INO4, INO2, OPI1	
9	SKN7, YAP1, TRR1, TRX2	
10	PHO4, CLN3, PHO85, CRZ1	
11	AFT1, SIT1	
12	IME1, IME2	
13	CBF4, MET4, MET30, MET32	
14	MIG1, HXK2	
15	RPN4, RPT2, RPT6, RPT3, RPT5, RPT1, SEM1, UBP6	

Networks 1–5 have at least one dosage compensation structure satisfying all topological constraints. For these networks, the transcription factor involved in the structure is **bolded**, the two network components in that structure are *italicized* and references documenting the interactions among those components and the TF are listed in the rightmost column. If a network contains more than one such structure, only one is shown.

We then checked if those 5 networks satisfied the stoichiometry requirement. For the GAL network, the Gal3p-Gal80p interaction was reported in the literature to be of 1:1 stoichiometry [20]. For the networks regulating pheromone response [11], and response to osmotic stress [12], we found evidence that both Gpa1p and Ptp3p has only one known MAPK-binding motif, whose mutation abolished MAPK binding [21,22], and mutations in a single amino acid residue in Fus3p and Hog1p were sufficient to abolish their binding to Ptp3p [12,21], indicating that they have only one binding site each for the MAPK-binding motif. Similarly, for the cell-cycle control network [13], mutation of two amino acid residues close together were sufficient to abolish Clb2p-Swe1p binding, suggesting that Clb2p only has a single binding site for Swe1p [17]. Finally, for the network regulating nitrogen catabolite repression [14], we found that, although Ure2p exists as a dimer in solution [23,24], Gln3p has only one domain that interacts with Ure2p [25].

## Discussion

Although dosage compensation characteristics of individual genes have received some attention over the years [7-9], we are not aware of any studies into the dosage compensation characteristics of entire gene networks, with the exception of the previous work [10] carried out by one of the authors of this manuscript. It is worth emphasizing that changes in gene dosage, whether by loss of a single copy of the gene, or by larger scale chromosomal structural changes, such as those that frequently occur in cancerous cells [26], typically result in changes in the dosage of some, but not all, genes in a gene network, to which network-dosage compensation does not apply. We expect changes in the effective dosage of entire gene networks to typically arise from far more mundane situations such as normal cell growth, global transcriptional variations, and other normal biological processes, but the very mundaneness of these situations is strongly indicative of the need for cells to be able to compensate for network-dosage changes. Thus, while it is perhaps unsurprising that most of the yeast genes are not compensated with respect to single gene dosage changes [8], our results here show that several gene networks with diverse biological functions carry the structure necessary for network-dosage compensation.

It should be noted that, while not all natural networks are expected to display the compensation requirements, our results are highly likely to underrepresent the actual number of natural networks satisfying such requirements. This is because, in cases where the “direct” activator or inhibitor in the network actually affects its own transcription via some intermediary protein, the network may well have the dosage compensation topology, but our method would not be able to detect them. In addition, our classification of genes into activators and inhibitors, necessary to detect the required network topology, is based on genetic interaction data and hence is necessarily limited by the availability of such data. In particular, as large-scale genetic interaction studies are frequently reliant on generation of double mutants [27], genetic interaction data are of relatively limited assistance with interactions among essential transcription regulators whose mutations can be lethal. Yet, those essential transcription regulators are likely the ones most in need of dosage compensation mechanisms. Thus, we believe that in reality the number of gene networks carrying the dosage compensation topology would likely be significantly larger.

## Conclusions

In summary, our work provides the most general network-dosage compensation analysis to date, expanding the analysis from 2-component gene networks to N-component networks. Here we show that the network-dosage compensation mechanism is not restricted to network components with specific gene expression and protein degradation levels, but the mechanism can robustly operate over large ranges. Furthermore, we show that the conditions that are necessary for an inducible network to be dosage compensated are also sufficient. Finally, using genome-wide binding and gene expression datasets, here we demonstrate the recurrent nature of the special topologies or motifs needed for network-dosage compensation. As a result of identifying and merging 82 compensation units (Additional file 4) in yeast, we obtained 15 larger networks (Table 2). Out of these 15 large networks, we were able to find evidence in the literature that 5 of them (33%) carried the dosage compensation requirements identified in our work (specific topologies and stoichiometry). This number, as we explain above, is likely to significantly underrepresent the actual number of dosage-compensated networks.

The dosage compensation motifs we analyzed show strong similarity to the sequestration-based regulatory networks [10,12]. Sequestration-based mechanisms are widely present in eukaryotic organisms, and regulate a wide variety of biological processes [28,29]. Dosage compensation is expected to be advantageous during natural selection. The widespread occurrence of sequestration-based mechanisms lends further support to the conclusion that network dosage compensation can be an important component of nature’s design for gene network architecture in cells.

## Methods

### Sampling of network parameters

All possible combinations of the parameters θ_a_, θ_i_, t_a_, t_i_ (144 in total, see Table 1) were tested for each network topology. For each combination of those four parameters and network topology, 10,000 sets of values for the parameters S_a_, S_i_, α and β were sampled from the corresponding distribution specified in Table 1, for a total of 1,440,000 networks examined per topology.

### Production of numerical inducibility curves

Each network is numerically integrated from t = 0 to t = 24 h for each possible value of g specified in the table. The starting state is assumed to be the steady state at basal transcription levels, i.e., $a=\frac{\theta_{a}\lambda_{a}}{\gamma_{0}+\gamma_{a}},i=\frac{\theta_{i}\lambda_{i}}{\gamma_{0}+\gamma_{i}}$. The value of *f(a,i,g)* at t = 24 h was calculated. A small percentage of the networks sampled displayed numerical problems during integration and were excluded from further analysis.

### Transcription regulators and transcription factor binding

We generated a list of all verified yeast ORFs annotated with the GO term “biological regulation” (GO:0065007) or one of its children using the Saccharomyces Genome Database [30]. For each regulator in the list, we obtained a list of transcription factors that bind to the promoter from the YEASTRACT database [31-33], limiting our search to documented evidence of TF binding to promoter. We also added regulator binding data from MacIssac *et al*., using moderate binding constraints and strong conservation constraints [34]. The original lists of regulators and transcription factors are provided in Additional files 2 and 3.

### Physical interactions

Physical and genetic interaction data for all genes involved were obtained from BioGRID [35]. To reduce false positives in physical interactions, we required two proteins to have either one reported low-throughput physical interaction or two reported high-throughput physical interactions in the database to be considered physically interacting.

### Classification of genetic interactions

Genetic interactions are classified into positive interactions and negative interactions. Positive interactions represent cases where the double mutant has a less severe phenotype than either single mutant, which indicates that the two network components should have different regulatory signs. Such interactions are classified in BioGRID as *synthetic rescue, positive genetic, dosage growth defect,* or *dosage lethality*. Conversely, negative interactions represent cases where a double mutant has a more severe phenotype than expected, and indicate that the two components in the gene network should complement each other, and therefore have the same regulatory sign. Such interactions are classified in BioGRID as *dosage rescue, negative genetic, synthetic growth defect, synthetic lethality,* or *synthetic haploinsufficiency*.

If the above system causes the overall interaction between two genes to be classified as both positive and negative, or if the only genetic interactions reported in BioGRID are classified as *phenotypic enhancement* and *phenotypic suppression* (the definitions of these terms are too broad to permit the simple classification above), then the interaction is manually classified based on the publications documenting the interaction.

## Availability of supporting data

The data sets supporting the results of this article are included within the article and its additional files.

## Competing interests

The authors declare that they have no competing interests.

## Authors’ contributions

MA conceived and guided the study, designed it with RS, and co-wrote the manuscript. RS participated in the design of the study, wrote the code for the simulations, performed the data analysis, and co-wrote the manuscript. PL contributed to the design and interpretation of the study. All authors read and approved the final manuscript.

## Supplementary Material

Additional file 1 This file contains supplementary Figures S1-S3. Click here for file

Additional file 2 List of the 1385 yeast regulators examined. Click here for file

Additional file 3 List of the 166 transcription factors that bind to the promoter of the yeast regulators examined. Click here for file

Additional file 4 List of the 82 PCUs. PCUs whose structures were verified to be consistent with dosage compensation are bolded. Click here for file
