# Supplementary material for: Network-dosage compensation topologies as recurrent network motifs in natural gene networks
Source: BMC Syst Biol. 2014 Jun 14;8:69. doi: 10.1186/1752-0509-8-69 (PMC4071340; doi:10.1186/1752-0509-8-69)
Supplement: Additional file 1 — This file contains supplementary Figures S1-S3. [file 1752-0509-8-69-S1.pdf]

Network-dosage compensation topologies as recurrent network motifs in natural gene networks

Ruijie Song[a,c], Ping Liu[b,c], & Murat Acar[b,c,#]

[a] Interdepartmental Program in Computational Biology and Bioinformatics

[b] Department of Molecular, Cellular and Developmental Biology

[c] Systems Biology Institute, Yale University, New Haven, CT 06511

[#] To whom correspondence should be addressed.

E-mail: murat.acar@yale.edu

Mailing Address: P.O. Box 27391, West Haven, CT 06516

Supplementary Information

Supplementary Figure Legends

Fig. S1. Quantification of inducibility and compensation penalties. **A.** Inducibility curves for N=1 and N=2 systems (containing one or two copies of the gene network) were obtained; the area between those curves is the penalty to network-dosage compensation. **B-C.** The inducibility penalty is the average of two metrics. The first metric (shown in part B) measures the overall range of the network output as compared to the reference curve. The second metric (shown in part C) measures how much the inducer level required to activate the network differs from the reference.

Fig. S2. Numerical analysis of the relationship between network-dosage invariance and network topology. **A-C.** Three topologies tested in the simulation (same as Fig. 1B-D) and their corresponding functional forms. **D-F.** For each network in **A-C**, a randomly-selected representative set of 20,000 networks (out of 1,440,000) were plotted; the penalty to dosage invariance was plotted on the X axis, while the penalty to inducibility was plotted on the Y axis. The green area represents networks that are both dosage invariant and inducible (both penalty <= 0.1). Yellow area indicates borderline networks (both penalty <= 0.15).

Fig. S3. Model parameters that do not significantly affect the network's ability to be dosage compensated. **A-C.** Histogram of the value of the sampled parameters S_a, S_i and α in dosage-invariant inducible networks having the topology of Fig. 1B (green area in Fig. S2D, N=6746). **D-F**. Histogram of the value of sampled parameters S_a, S_i and β in dosage-invariant inducible networks having the topology of Fig. 1C (green area in Fig. S2E, N=6124).

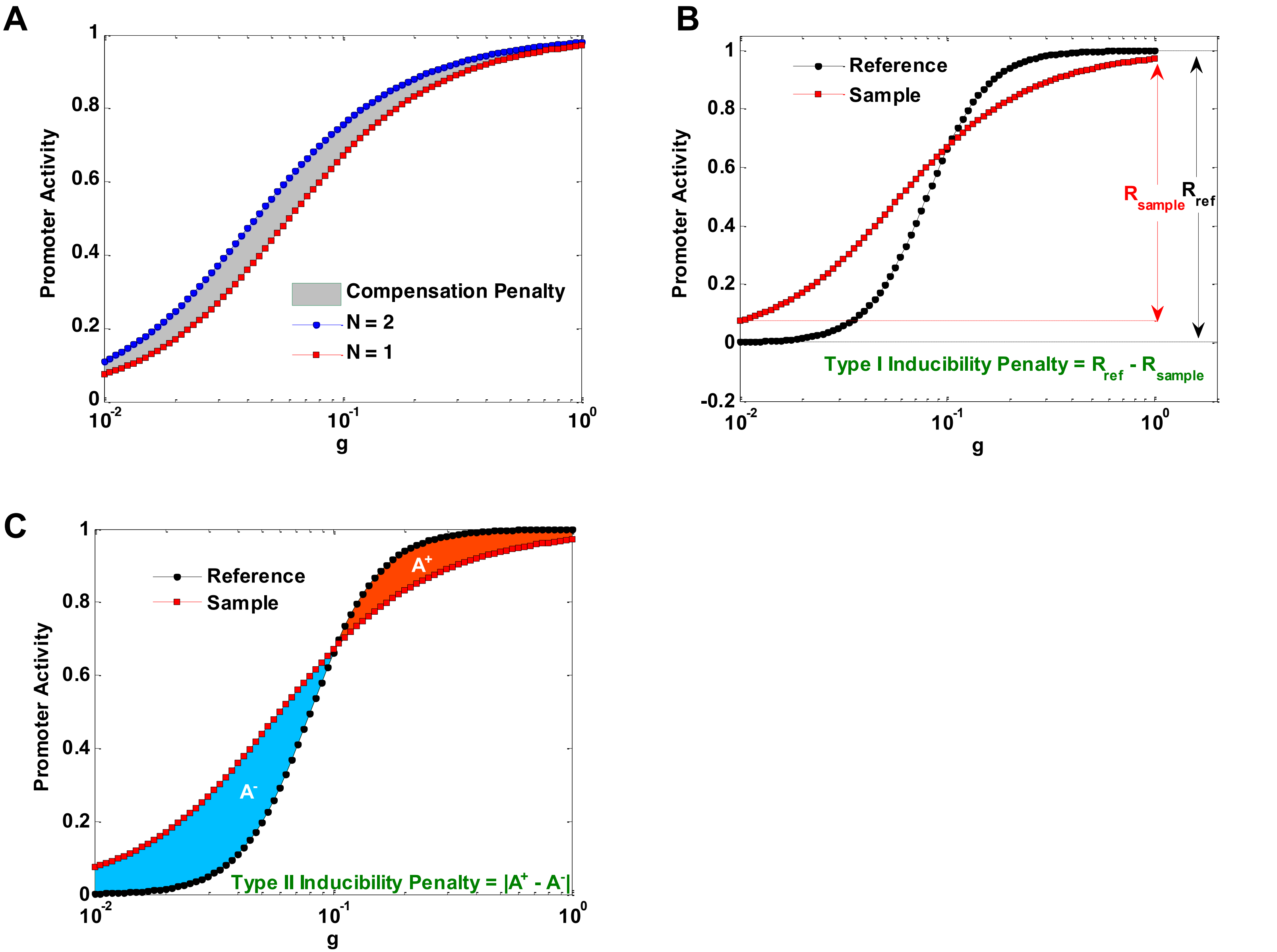
A
Promoter Activity
Compensation Penalty
N = 2
N = 1
g
B
Reference
Sample
R_{sample}
R_{ref}
Type I Inducibility Penalty = R_{ref} - R_{sample}
C
Reference
Sample
A^+
A^-
Type II Inducibility Penalty = $|A^+ - A^-|$

Fig. S1

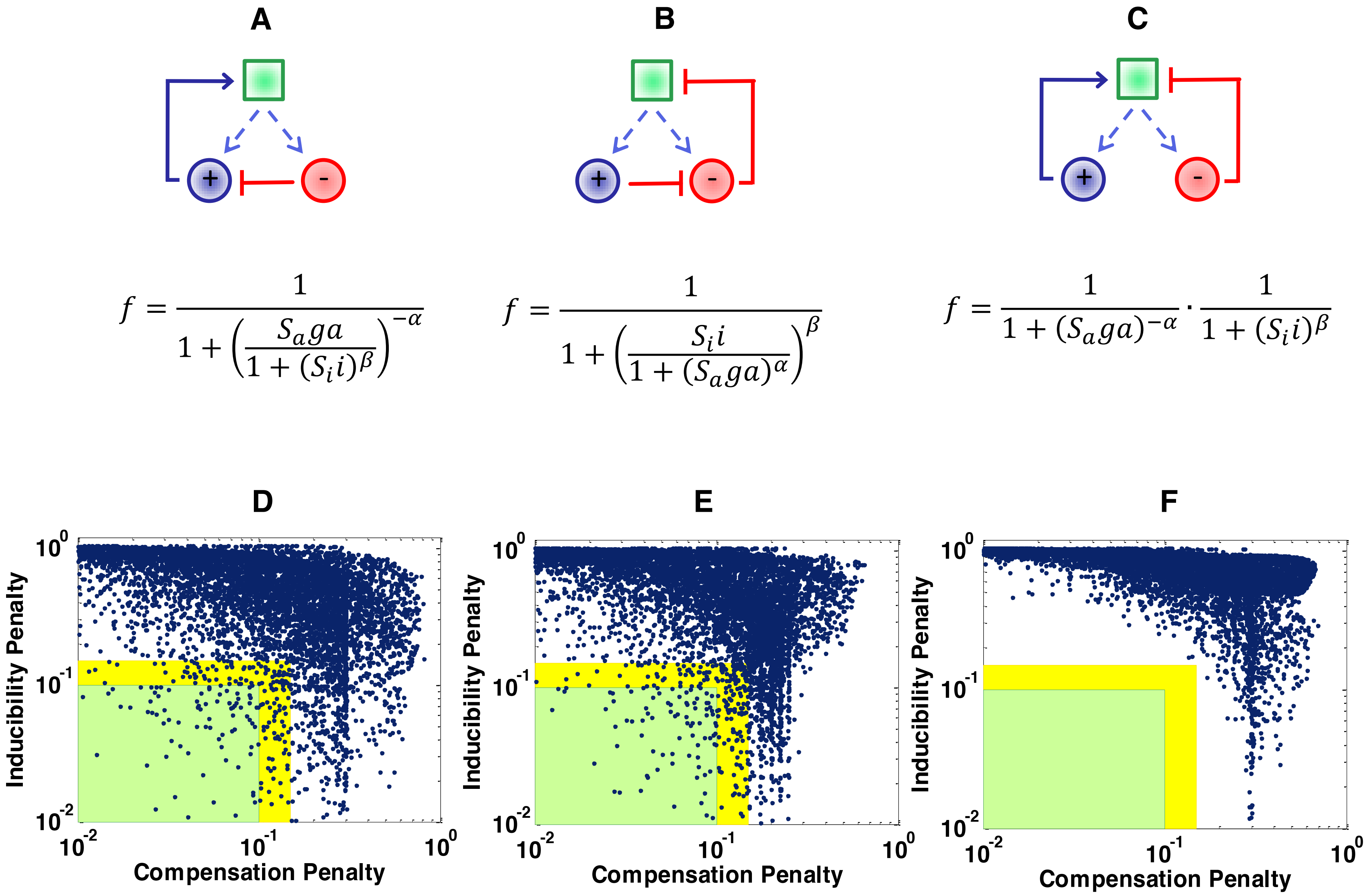

A
B
C
+
-
$f = \frac{1}{1 + \left(\frac{S_a g a}{1 + (S_i i)^{\beta}}\right)^{-\alpha}}$
$f = \frac{1}{1 + \left(\frac{S_i i}{1 + (S_a g a)^{\alpha}}\right)^{\beta}}$
$f = \frac{1}{1 + (S_a g a)^{-\alpha}} \cdot \frac{1}{1 + (S_i i)^{\beta}}$
D
E
F
Inducibility Penalty
Compensation Penalty
10^{0}
10^{-1}
10^{-2}

Fig. S2

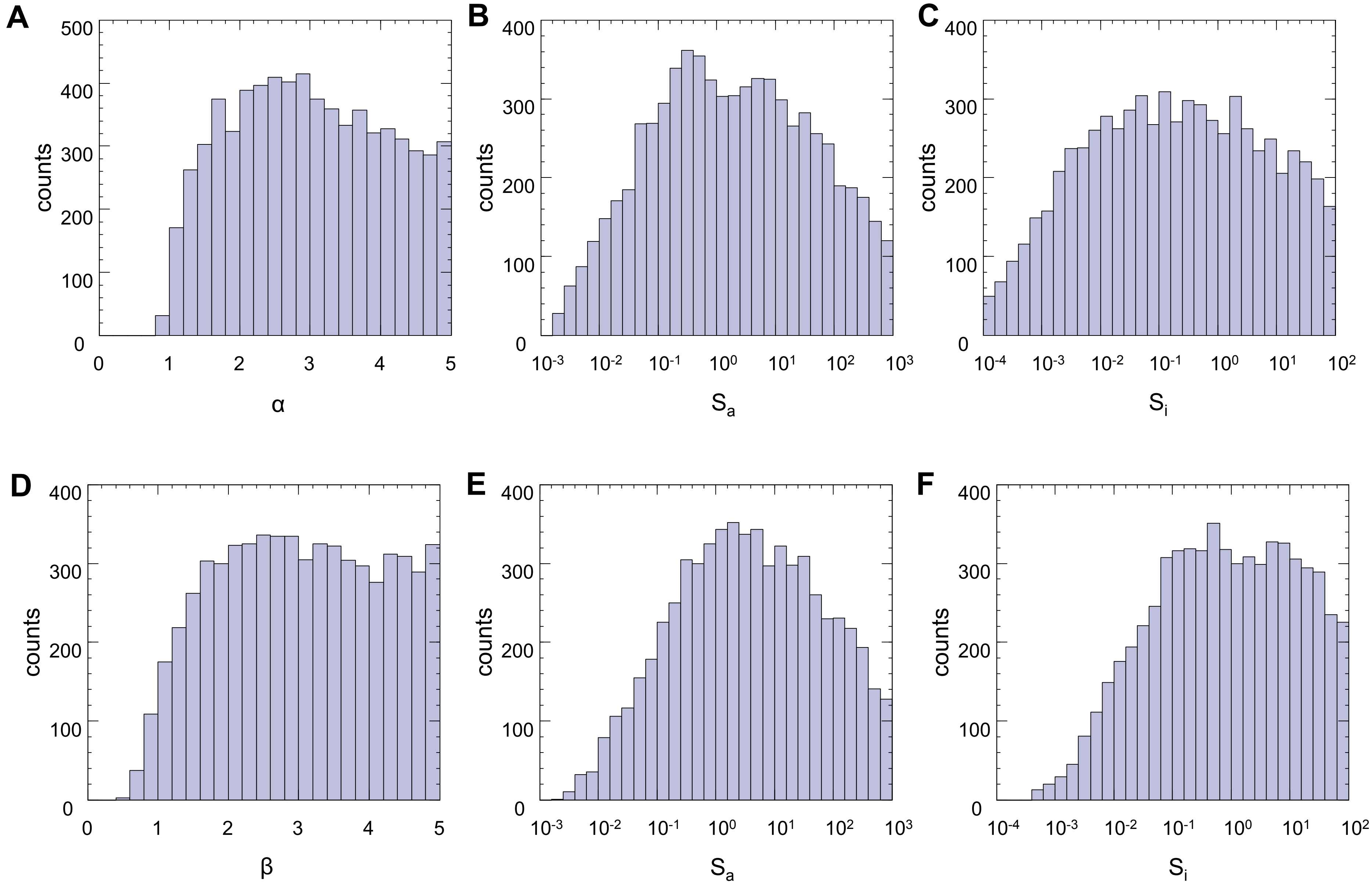

Fig. S3
